# Single-cell adhesion force kinetics of cell populations from combined label-free optical biosensor and robotic fluidic force microscopy

**DOI:** 10.1038/s41598-019-56898-7

**Published:** 2020-01-09

**Authors:** Milan Sztilkovics, Tamas Gerecsei, Beatrix Peter, Andras Saftics, Sandor Kurunczi, Inna Szekacs, Balint Szabo, Robert Horvath

**Affiliations:** 1 Nanobiosensorics Group, Institute of Technical Physics and Materials Science, Centre for Energy Research, Budapest, Hungary; 2 Department of Biological Physics, Eötvös University, Budapest, Hungary

**Keywords:** Cellular motility, Imaging and sensing

## Abstract

Single-cell adhesion force plays a crucial role in biological sciences, however its in-depth investigation is hindered by the extremely low throughput and the lack of temporal resolution of present techniques. While atomic force microcopy (AFM) based methods are capable of directly measuring the detachment force values between individual cells and a substrate, their throughput is limited to few cells per day, and cannot provide the kinetic evaluation of the adhesion force over the timescale of several hours. In this study a high spatial and temporal resolution resonant waveguide grating based label-free optical biosensor was combined with robotic fluidic force microscopy to monitor the adhesion of living cancer cells. In contrast to traditional fluidic force microscopy methods with a manipulation range in the order of 300–400 micrometers, the robotic device employed here can address single cells over mm-cm scale areas. This feature significantly increased measurement throughput, and opened the way to combine the technology with the employed microplate-based, large area biosensor. After calibrating the biosensor signals with the direct force measuring technology on 30 individual cells, the kinetic evaluation of the adhesion force and energy of large cell populations was performed for the first time. We concluded that the distribution of the single-cell adhesion force and energy can be fitted by log-normal functions as cells are spreading on the surface and revealed the dynamic changes in these distributions. The present methodology opens the way for the quantitative assessment of the kinetics of single-cell adhesion force and energy with an unprecedented throughput and time resolution, in a completely non-invasive manner.

## Introduction

Understanding cellular adhesion is of great importance for life sciences as the majority of cell types have to adhere to surfaces in their environment in order to survive and to take part in the vital processes of tissue development and reorganization, cell motility, immune response and others^1^. This substantially complicated process involves a multitude of factors both on the outside and the inside of the cell membrane, such as membrane-bound adhesion proteins^2^, glycocalyx elements^3,4^ and the cytoskeleton^5^. Adhesion under *in vitro* conditions progresses through passive adsorption to the surface (at this level the initial contact will be made by the cell glycocalyx coat), attachment, spreading and the formation of focal adhesions while under *in vivo* conditions it is further modulated by flow circulation^6^, signalization processess^7,8^ or extracellular matrix components. Cells express a wide range of adhesion receptors that bind the same or different ligands with varying affinity^9^. The strength of adhesion strongly depends on how long the cell is allowed to adhere to a substrate (the number of integrin-ligand pairs and consequently, the overall contact area increase with time), on substrate rigidity, lateral spacing of the ligands^10^ as well as on ligand tether length. In addition to integrins, the glycocalyx, consisting of glycoproteins, glycolipids, proteoglycans and polysaccharides, can also be involved in the cell adhesion process. Cell adhesion research provides important knowledge for the development of tissue-on-a-chip^11,12^ and organ-on-a-chip^13,14^ biosensors for tissue engineering, as well as for studying cancer progression and its treatment therapy. Over the years, numerous methods have been introduced to examine and quantify cell adhesion, from simple observations in an optical microscope to increasingly elaborate atomic force microscopy (AFM) techniques^15–19^. These techniques either measure cell-surface interactions and cell adhesion kinetics^10,20–24^, or they are based on applying a force that can lead to cell detachment (termed adhesion strength measurements) on single-cells (e.g., micropipette aspiration, AFM, optical tweezer techniques) or on cell populations (e.g., centrifugation assay, spinning disk, flow chamber)^25^. One critical parameter of cell adhesion measurement methods is the throughput, describing the number of cells that can be detached in a certain period of time^26^. Since single-cell force spectroscopy methods work with one cell at a time, their throughput is limited and can hardly be used to account for single-cell variability. The most common of such cell detachment methods is force spectroscopy performed by an AFM machine, which uses functionalized cantilevers to first grab a cell, let it adhere to the surface, then detach it. By varying the time of contact, such an arrangement can provide information about the kinetics of adhesion between a live cell and a substrate (or another cell) as demonstrated by Strohmeyer *et al*.^27^. This method can be beneficial for molecular level investigations, however, it is not ideal to detach fully spread out and adhered cells. Moreover, every force curve measurement requires a separate cantilever that needs to be functionalized and calibrated, which makes the method particularly slow, capable of measuring a single-cell detachment event in a matter of hours^28^. Furthermore, the adhesion kinetics of individual cells remains hidden, since only a particular time instance of the adhesion process can be examined. An additional issue is the effect of the cantilever which is fixed to the top of the cell, adding an undesired element to its microenvironment.

**Figure 1:**
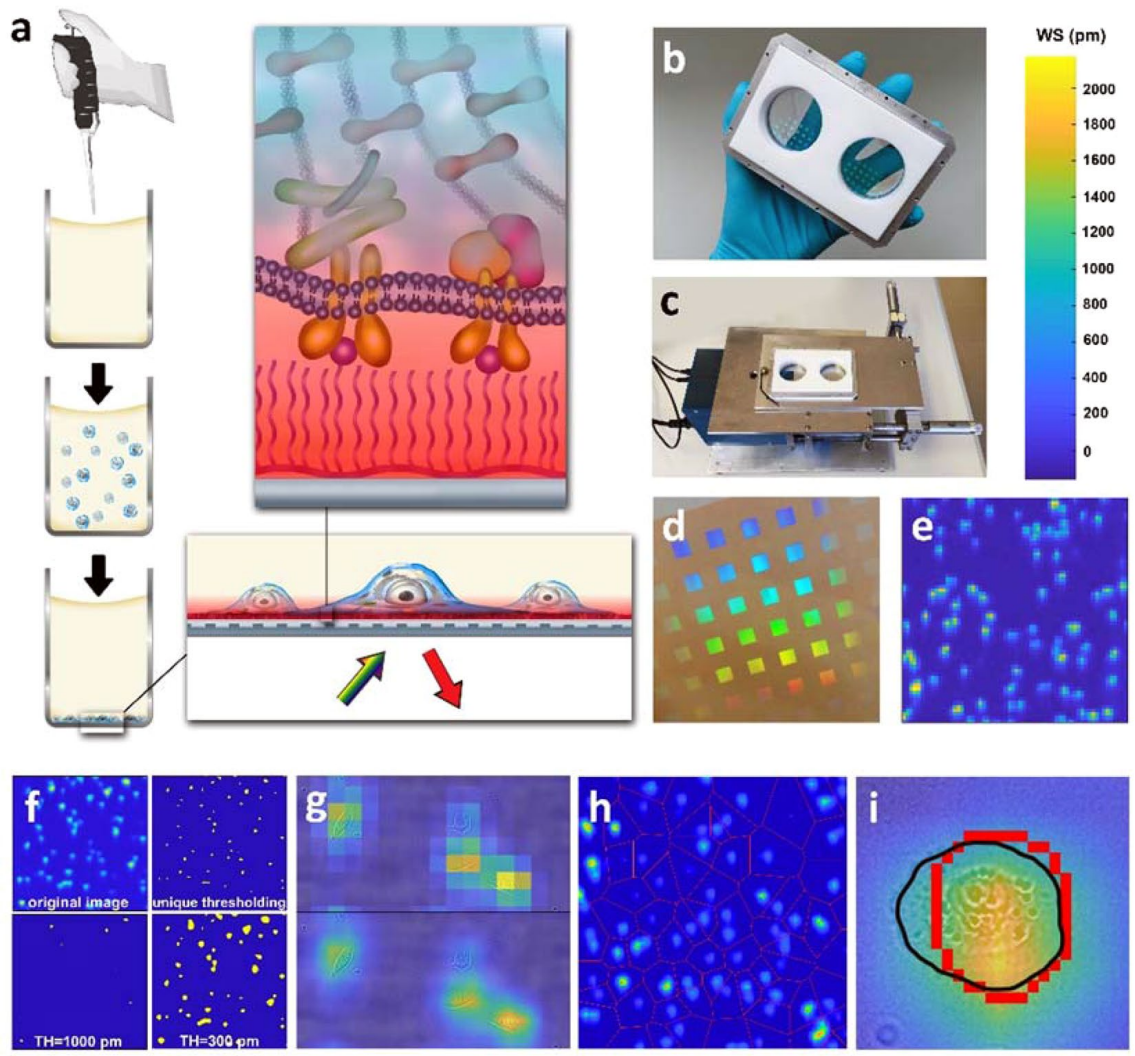
The optical biosensor measurement workflow and results. (**a**) Schematic of the measurement workflow. The cancer cells are pipetted into the custom well containing the array of 2 × 2 mm optical sensors. After sedimentation cells adhere to the functionalized sensor surface that is illuminated from below (yellow-green arrow) and reflects only a certain resonant wavelength (red arrow). The evanescent field (red shadow above the sensor) penetrates into the surface structures of the cell such as the integrins, the membrane, the actin filaments and the additional proteins that make up the adhesion site (top right drawing). (**b**,**c**) Photographs showing the custom-made biosensor insert holder (in a hand, and placed into the Epic Cardio device) with two circular wells optimized for subsequent FluidFM BOT measurements. The large area of each well contains twelve 2 × 2 mm^2^ biosensors simultaneously read out, and allows for navigating later with the FluidFM BOT probe over centimeter wide surfaces. (**d**) Photograph of the Epic Cardio biosensor insert. The 2 × 2 mm^2^ sensor areas are visible as colorful squares due to light diffraction on the embedded grating. (**e**) Raw WS signal image of a single sensor area at t = 90 min (color bar at top right corner). Individual cells are well separable as the pixel size is 25 × 25 µm^2^. (**f**) Comparison of different thresholding strategies of recorded biosensor images. The top left part shows the original biosensor image with a 3^rd^ degree interpolation. The bottom left and right pictures show the effect of applying a constant threshold of 1000 and 300 picometers respectively: the former underestimates the cell areas, while the latter overshoots and creates unrealistic interconnected cells. It is apparent that the introduced unique thresholding (top right image) gives a better agreement with the original image and the cell perimeter can be determined accurately. (**g**) Fused image of the biosensor signal and the brightfield picture, showing a clear correspondence between the two overlapping modalities. Moreover, it is observable that cells with similar area can produce distinct WS amplitudes, thus our IWS (incorporating both size and WS of the cell) is indeed a suitable measure of cell adhesion. On the upper part, the original resolution biosensor data was used, while the lower image is a 4^th^ degree interpolated picture. (**h**) The Voronoi tessellation of a sensor area. Red dots indicate the local maxima of the signal that were taken as the generators of the tesselation. Red edges separate the individual segments belonging to the generator included within them. During evaluation, a cell-specific threshold calculated from the maximal pixel value was applied individually in every segment. (**i**) Area matching segmentation: the combined optical biosensor and brightfield picture shows how the segmented cell perimeter (red) approximates the actual cell perimeter measured on the microscope image (black) after setting the optimal threshold.

So far, the most successful tool to tackle the challenge of exploring the real-time kinetics of cell adhesion has been the application of surface sensitive label-free methods (surface plasmon resonance (SPR)^29,30^, infrared SPR^31–33^, photonic crystals^34–36^, quartz crystal microbalance^37–39^, ellipsometry^40,41^, digital holographic microscopy^42,43^), most of them are using surface bound evanescent waves. For example, the resonant waveguide grating (RWG) based optical biosensors measure the local refractive index change in the 150 nm vicinity of the sensor surface, thus providing real-time kinetic information on cell adhesion and spreading, typically on a population of cells (Fig. 1a). The sensor itself is an optical grating incorporated into the support of the high refractive index (RI) waveguide layer. The grating is illuminated from below and reflects only a narrow wavelength band of the incoming light depending on the local refractive index in the evanescent field. In case of an adhering cell the evanescent field overlaps with the cell-substrate contact distance (10–80 nm)^44^, the cell membrane (5–10 nm) containing the integrins and other transmembrane proteins, the protein complexes linking the intracellular domain of the integrins to the actin fibers (6–7 nm)^45^ such as the Arp2/3, vinculin, VASP and finally part of the actin cytoskeleton itself. Because of the very limited penetration depth of the evanescent field, cell organelles that are irrelevant to adhesion do not contribute to the signal thus the information collected from the cell-substrate area is indicative of the adhesion process^10,20^.

This novel method has been used to examine the behavior of live cells on biofunctionalized surfaces with extremely high sensitivity and throughput^23,46–48^. The possibility to chemically modify the sensing surface allows for the examination of specific molecular interactions, such as Arg-Gly-Asp tripeptide (RGD motif) mediated activation and clustering of integrin proteins. The high-quality data provided by this technique have found its use in various fields such as receptor biology^49–51^, immune cell biology^52^, environmental toxicology^53^, or the testing of natural active compounds^22,54–56^. Depending on their configuration, RWG sensors are generally compatible with standard 96- or 384-well plates, providing a platform for high-throughput measurements, where numerous cell populations can be measured at the same time under different conditions.

In recent years, however, the emphasis of experimental biology has been shifting from averaging over populations (as in the traditional RWG measurements) to acquiring biological information directly from single-cells^57^. This paradigm shift has been fueled among others by drug discovery^58,59^, cancer research (such as circulating tumor cell (CTC) studies)^60,61^ and the development of computational tools necessary for interpreting the massive amount of data emerging from such measurements^62,63^. This rapid progress calls for new tools capable of measuring cell-surface interactions with high-throughput and single-cell resolution. The challenge is potentially addressed by novel high spatial resolution optical biosensors capable of recording real-time data from thousands of individually resolved cells in parallel^34,47,55,64^. This unrivaled throughput and the possibility to conduct experiments in physiological conditions (e.g. in an incubator) makes these devices exceptionally powerful tools for single-cell level adhesion biology research. In the present study we employed the novel RWG-based Epic Cardio biosensor system (see Fig. 1c) with 25 micron spatial resolution^55^. It is important to note that the spatial resolution of the technique can be conveniently increased by improving the camera and optics inside the device, creating the possibility of measuring the adhesive properties of cells on a sub-cellular level.

While the RWG biosensor signal is known to describe the mass redistribution of cells on the sensor surface, thus characterizing adhesion and spreading, it has never been directly compared to actual adhesion force or adhesion energy values as measured directly by an external method on individual cells. The assumption is that the biosensor signal correlates with the adhesion force of live cells, since it is proportional to the amount of proteins near the cell surface that are responsible for the formation of cell-substrate bonds. For testing this hypothesis one needs to directly measure the adhesion force of cells attached to the functionalized biosensor surface and compare the values to the recorded biosensor signal (wavelength shift (WS)) of the same cells. In order to execute this measurement, a tool capable of recording single-cell force curves over a relatively large surface area with high accuracy is needed. Considering the RWG system employed here, the dimensions of the optical sensor surface on which the cells are attached is 2 mm × 2 mm, twelve such sensors are placed in an array with a distance of 2 mm in between, making it necessary to cover an area of minimum 2–3 cm^2^.

In order to reach the whole sensor surface, robotic fluidic force microscopy (FluidFM BOT), a novel AFM-based force spectroscopy tool was used. This technique allows for the positioning of the probe over large, mm-cm scale areas^65^. The force measurement is executed according to the AFM principle and a fluidic channel with a circular aperture incorporated into the cantilever is responsible for fixing the cell to the end of the cantilever^18,66–69^. Applying this method, we are able to mechanically separate adhered cells directly from the biosensor surface and measure the corresponding force-distance curves that can be evaluated for extracting maximal adhesion force and adhesion energy values.

In this study we introduce a combined experimental arrangement to calibrate the surface sensitive label-free optical biosensor signal using direct force spectroscopy measurements on a large number of mammalian cells. Once the relationship between the measured optical signal and the adhesion force is established for the first time, we are able to record the real-time adhesion force kinetics of more than 300 cells over a period of 1.5 hours.

## Results

### Combined optical biosensor - robotic fluidic force microscopy measurements on single-cells

In order to measure the adhesion of several hundred cells in a label-free, non-invasive manner with a high temporal resolution, we applied the RWG-based Epic Cardio biosensor^46,55^. HeLa cells were deposited on the sensor functionalized using poly(L-lysine)-*g*-poly(ethylene-glycol) polymer grafted with RGD motifs (hereafter PPR) to facilitate adhesion (Fig. 1a). The RGD motifs serve as adhesion initiators for the HeLa cells since they express RGD-specific α_IIb_β_3_, α_v_β_3_ (overexpressed) and α_5_β_1_ integrins^70,71^. When cells are in suspension, the integrins are distributed randomly on the cell surface. Approaching surface-bound RGD ligands, integrins form clusters and generate focal adhesions. The cells were let to adhere for 90 min during which their adhesion kinetics showed a saturation signal.

**Figure 2:**
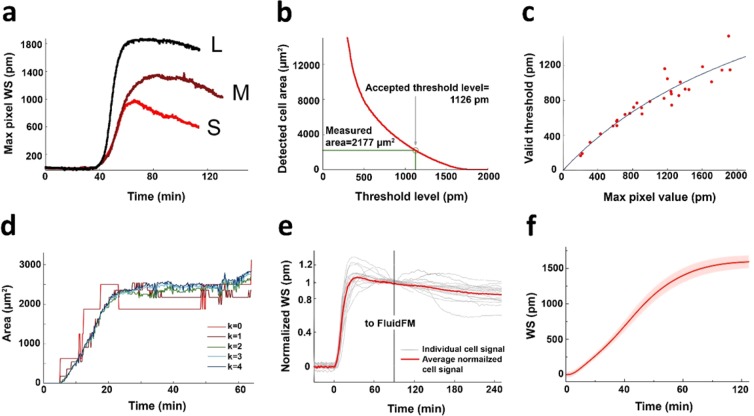
(**a**) Resonant wavelength shift of the pixel with the highest signal in case of 3 different cells (Large, Medium and Small spread area cell) as a function of time. (**b**) Dependence of the cell area deduced from the biosensor data on the threshold level. The threshold is accepted where the corresponding area matches the one measured using the brightfield image. (**c**) The relationship between the maximal pixel value of the cell-covered area and the corresponding adequate threshold level. The latter is defined as the threshold value that separates the cell from the background on the biosensor image in such a way that the area of the cell will match the area measured on the brightfield microscopy image. The blue line shows the fitted saturation curve based on which individual cell threshold levels are calculated for further cells. (**d**) The effect of interpolation degree on the area of a single-cell: it is apparent that interpolation does not affect the general dynamics of the area evolution, it only smooths it due to the finer spatial resolution. (**e**) Data showing the stability of the wavelength shift (WS) signal during a measurement time (t) of 270 minutes from the point when cells were added to the biosensor well (t = 0 min). The first 60 minutes shows the spreading kinetics of the cells measured by the biosensor in real-time. During the combined measurement the plate is placed into the FluidFM BOT at t = 90 min and the force-distance curves are recorded for around 2 hours. The relative stability of the signal (10% shift/120 min) demonstrates that the cells are not considerably damaged during the FluidFM BOT experiment. The signal is normalized to the value at t = 90 min, the time of placing the custom biosensor insert holder to the FluidFM BOT instrument. (**f**) Typical adhesion kinetics of a cell population averaged over the sensor area (2000 HeLa cells deposited on RGD-grafted PLL-g-PEG surface). The shadow represents standard deviation from three parallel experiments.

The sensor itself as seen in Fig. 1b,c consists of a transparent high refractive index waveguide layer with an incorporated grating. The primary signal of the sensor is the shift in the resonant wavelength of the incoupling light, which is given in picometers and is commonly referred to as dynamic mass redistribution (DMR) signal in the literature^47^. The sensitive surface is a 2 mm × 2 mm zone on the waveguide plate into which the resonant grating is embedded (Fig. 1d). In the standard plate arrangement, every sensor is placed on the bottom of a well of a 384-well biosensor microplate. Each well can be filled with the sample solution containing approximately 2000 cells, depending on the cell type. If the signal is averaged over the sensitive area, the kinetic curve shows a distinct sigmoid shape (Fig. 2f) whose parameters characterize the extent and rate of cell adhesion, providing valuable biological information.

Using a high spatial resolution redout, the WS signal is collected from a smaller segment of the surface, which is represented by a pixel. Instead of averaging over the whole sensor area, when we look at a sole pixel belonging to a cell, kinetic curves such as the one in Fig. 2a can be extracted. By plotting the pixel values in an image format, we can acquire images such as the one in Fig. 1e. Frames are recorded every 3 seconds following the kinetics of the WS in real-time.

To fully characterize cell adhesion our aim was to collect information from every pixel that can be considered to belong to a given cell. In order to do so, we devised a method for the adequate analysis of the recorded images. Our method is based on interpolation, segmentation and a subsequent thresholding that combines the biosensor data with brightfield microscopy images for achieving maximal accuracy.

First, we define the integrated wavelength shift (IWS) of a given cell on a given frame as:

1 $$IWS={A}_{pix}\cdot \mathop{\sum }\limits_{i:WS > threshold}^{\,}W{S}_{i}$$

Where A_pix_ is the area of a single pixel, and WS_i_ is the wavelength shift signal of a given pixel. The summation goes over the pixels (with indices “i”) that represent the area covered by the investigated cell. In order to distinguish the cells from the background, we define a cell-specific threshold value (noted as ‘threshold’ in Eq. 1), above which the pixels are considered to be constituents of the cell-covered biosensor area. Therefore, only such pixels are included in the summation whose WS value is above the threshold (WS > threshold). The valid threshold is defined in such a way that upon application, the overall area of the above-threshold pixels should match the area deduced from the corresponding brightfield optical image (Fig. 1g,i). Determining the threshold this way ensures that no unrepresentative data from the background are included in the integration of the IWS values. The dimension of the defined quantity is area × wavelength, our unit of choice is µm^2^ × pm. IWS incorporates two processes which fundamentally determines the strength of adhesion: recruitment of cell adhesion molecules (CAMs), as their density is proportional to the WS, and cell spreading, which increases the area available for recruitment.

However, the spatial resolution of the Epic Cardio device used in our experiments is 25 µm, which is inadequate to robustly determine the perimeter of the cells. To solve this problem a linear interpolation of the two dimensional WS data was applied, reducing the size of the pixels. The effect of interpolation degree on the cell area was examined and is presented in Fig. 2d. The data suggests that higher interpolation degrees decrease the fluctuation in area (and jointly in IWS), that is inherently caused by the relatively large pixel size. Once the interpolated pixel size is reasonably small compared to the area of the cell, further interpolation has negligible effect on the magnitude of the signal. Due to this effect an interpolation degree of k = 4 was used, adjusting the interpolated pixel size to 1.7 µm, providing a smooth time dependence of IWS signal.

Following the interpolation, the corresponding threshold level of 30 cells was determined from the combination of brightfield microscopy and biosensor images. If we plot the acquired threshold values as a function of the maximal WS (WS_m_) value in the corresponding cell, a pattern emerges as seen in Fig. 2c. The data indicates that there is a clear relationship between the value of the adequate threshold (AT) (the one that gives a cell area in accordance with the brightfield microscopy images) and the maximal WS signal of the cell. The relationship is best fitted with a simple saturation curve in the form of

2 $${\rm{AT}}=\frac{{\rm{a}}\cdot {{\rm{WS}}}_{{\rm{m}}}}{{\rm{b}}+{{\rm{WS}}}_{{\rm{m}}}}$$

where the fitted parameters were determined to be a = 3128 pm and b = 2935 pm with R^2^ = 0.884 characterizing the goodness of fit. Importantly, this correlation offers the possibility to determine the threshold values of further cells detected without the need to record their optical microscopy images.

Since a unique threshold belongs to each cell, a segmentation of the biosensor image is required to assign a specific environment to the cells in which the cell-specific threshold can be applied. To this end, we created an algorithm that finds the local maxima of the biosensor images, then applies a Voronoi tessellation to the image assigning segments to each cell (Fig. 1h). The local maxima in the 2D signal are considered to be cell centers and they are used as the generators to the Voronoi partitioning, which separates the plane into such regions that every point within is closer to the generator contained in the region than to any other generator. A unique threshold deduced from the maximal pixel value is applied in every segment, then the IWS value is determined by integration over the above-threshold pixels. We found this method to be superior to simple cut-off thresholding as it can identify cells in an extremely robust way (Fig. 1f).

Using this protocol, we could define an optimal IWS value for each cell which characterizes the adhesion strength while making use of the spatial resolution of the sensor. The segmentation and thresholding algorithm makes sure that the background is adequately separated from the areas containing the cells. The whole evaluation method was automatized in a MATLAB pipeline code that processes raw, whole-sensor WS data and outputs single-cell IWS time series.

**Figure 3:**
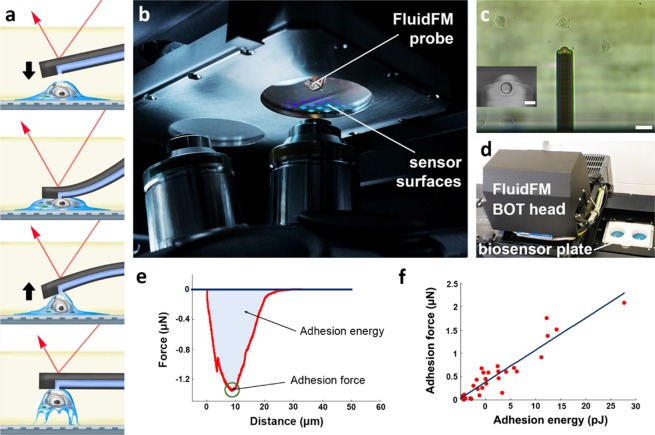
Principle and results of the robotic fluidic force microscopy (FluidFM BOT) measurements. (**a**) Workflow of the cell adhesion strength measurement using the FluidFM method. The hollow cantilever is approached to the adhered cell (top frame). Upon contact, vacuum is applied to fix the top of the cell to the cantilever (middle frame). Once a stable connection is established, the cantilever is retracted while its bending is recorded by the reflected laser beam deflection (red arrow). (**b**) The custom-made biosensor insert holder positioned in the sample holder of FluidFM BOT device. The photograph is made from below showing the objectives of the inverted microscope of the FluidFM BOT, the two circular wells, the sensor surfaces (reflective colored squares in the right well) and the probe on the upper side of the sensor insert. (**c**) Live view image of the fluidic force microscopy measurement: the picture shows the cantilever (and the purple laser spot on it) with surrounding adhered cells (scale bar: 40 *μm*). Inset: scanning electron microscopy image of the top of the cantilever. The aperture with an 8 µm diameter can be clearly seen. Scale bar: 8 *μm* (Image provided by Cytosurge AG). (**d**) Photograph showing the FluidFM BOT device on an anti-vibration table. The stage is fixed on an inverted microscope with the head unit above the objective. The inserted biosensor plate is seen at the right. (**e**) A typical force-distance curve of a measured cell and its evaluation. The adhesion energy is given by integrating the area under the curve from the point of contact (blue area). The adhesion force is defined as the maximal value of the force exerted by the cell-surface contact to the cantilever (green circle). (**f**) Plot showing the correlation between the adhesion force and adhesion energy values of the cells measured in our fluidic force microscopy experiments. While the two values both characterize the strength of the adhesion, they can represent different biological information.

In order to calibrate the above described IWS signal originating from the interaction with the evanescent field, we used a novel robotic fluidic force microscopy (FluidFM BOT) setup capable of measuring the force curves of individual adhered cells during their separation from a surface^18,67,72^. After cell adhesion, the custom-made sensor plate (Fig. 1b) was taken out of the biosensor instrument (Fig. 1c) and placed into the sample holder of the FluidFM (Fig. 3b,d). Using scratch marks introduced into the sensor surface before the measurement, we identified the position of cells that gave a clear biosensor signal, and proceeded to detach those cells from the surface, recording their force-distance (FD) curves.

The principle of the fluidic force microscopy measurement is shown in Fig. 3a. The adhered cell is approached by a hollow cantilever with an incorporated channel inside. The channel ends in a circular aperture (d = 8 µm) on the down facing side of the cantilever (Fig. 3c inset) while the other end is connected to a pressure controller unit which is capable of varying the pressure between 1000 and −700 mbar. Upon contact with the cell, a negative pressure is applied, effectively fixing the upper membrane of the cell to the aperture on the downward side of the cantilever. Once the contact is established, the cell is slowly pulled up, separating it from the surface. The aperture size and the maximal value of the negative pressure determines the force that fixes the cell to the cantilever, thus the highest force that can be measured. Using our experimental arrangement, this force is F_max_ = 3.5 µN. If the cell has a higher adhesion force, the contact between the cell and the cantilever will break up before the cell can be detached. Typically, HeLa cells adhered to the RGD-displaying surface used in our experiments have a lower adhesion force, up to around 2 µN. The bending of the cantilever during the process is measured using a laser beam reflected from its upper surface, corresponding to the AFM principle. The operator has the possibility to define the retraction speed, the applied vacuum, the time of contact as well as the setpoint, which is the desired contact force exerted on the cell during approach. The bending of the cantilever is calibrated to a force value using the spring constant of the cantilever (as measured by the Sader method^73^) and the inverse optical lever sensitivity.

Finding the correct settings is vital for conducting experiments with live cells, due to their mechanical fragility and sensitivity to environmental factors. A critical parameter is the loading rate of the detachment, which represents the rate at which the force is applied on the cell while being pulled up by the cantilever. This quantity (commonly measured in nN/s units) determines the measured detachment force in a way that has been extensively studied in dynamic force microscopy for the case of single^74^ and multiple bonds^75^. However, live cells represent a more complicated system due to the variability of their surface connections and their viscoelastic properties^76^. Since not only the molecular cell-surface bonds are unbinding in a loading rate-dependent manner, but also the overall mechanical properties of the cell depend on the pulling speed, it is crucial to keep this parameter constant during the measurements. In the experiments presented here, the retraction speed was set to 1 µm/s, which represents a loading rate in the range of 2–3 µN/s, given the typical measured spring constant of 2–3 N/m. In our experience, using such a loading rate is optimal for separating the cells from the surface in a non-destructive way, while maintaining the contact with the cantilever.

During detachment, the force-distance curves of the cell separation from the surface can be recorded and adhesion force and energy values can be extracted using the standard method accepted in the literature^77^ (Fig. 3e). The viability of cells prior to targeting was indicated by their visible morphology and their adhesion curve measured by the RWG sensor^78^. Survival following detachment was not investigated. A measurement was only accepted if there was no remaining debris on the surface after detachment (see Supplementary Information, Fig. S1a,b), since such remains would imply phyiscal damage to the cell membrane (Fig. S1c,d)^79^. Another prerequisite for a succesful measurement was the cantilever bending signal returning to baseline level.

We executed the combined biosensor-FluidFM measurement for 30 cells altogether, determining their IWS value as defined previously from the optical biosensor image and the corresponding adhesion force and energy values from the FluidFM BOT measurement (Fig. 3). For validation, long-run experiments were carried out to check if the IWS signal is stable enough throughout the subsequent force measurements. Based on the data presented in Fig. 2e the WS appears to be stable for at least 2.5 hours after moving the plate holder into the FluidFM BOT, which means that the cells did not suffer considerable degradation during the time of the detachment experiments.

**Figure 4:**
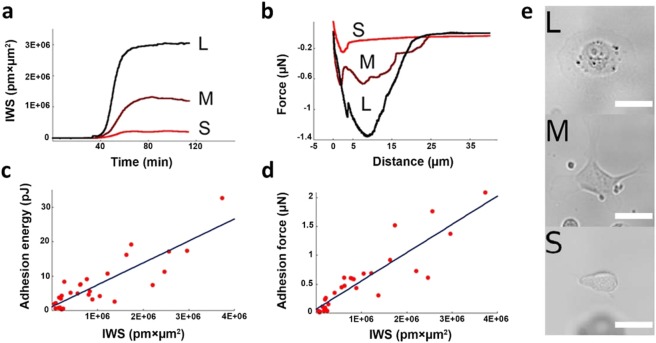
Calibration of the biosensor signal by FluidFM BOT measurements (**a**) Demonstration of the developed methodology: Individual cells are first let to spread and adhere in the biosensor device while their IWS signal is recorded. (**b**) Afterwards, the same single-cells are detached from the surface and their force-distance curves are measured using the FluidFM BOT device. (**c**,**d**) Correlation between the IWS signal and adhesion energies as well as IWS signal and adhesion forces measured in the experiments on the same single-cells. The linear correlation coefficients were determined as C = 0.8607 for the adhesion energy- and C = 0.8945 for the adhesion force versus the IWS recorded by the optical biosensor. (**e**) Corresponding brightfield microscopy images are also taken in order to facilitate the thresholding of the Cardio imager data. The scale bars represent 100 µm. (S, M and L marks a typical Small, Medium and Large sized cell, respectively.)

By comparing the data measured by the FluidFM BOT and the Epic Cardio biosensor on single-cells we see a clear correlation with a correlation coefficient of C = 0.8607 for the adhesion energy and C = 0.8945 for the adhesion force (Fig. 4c,d).

Since single cells are extremely complex systems, there is considerable variation in their behavior even in the exact same experimental conditions. This phenomenon means that while by measuring a large number of cells such variations are averaged out in the correlation parameters, calculated values for individual cells might exhibit relatively large deviations.

Nonetheless, the relationship between the optical signal and mechanical adhesion parameters can be fitted well with linear functions where the slopes are (4.9 ± 0.5) × 10^−4^ nN/(pm·µm^2^) for the adhesion force and (6.4 ± 0.7) × 10^−6^ pJ/(pm·µm^2^) for the case of adhesion energy. This result strongly supports the working hypothesis that the optical signal measured by the RWG sensor is a useful characteristic of the adhesion strength of live cells and it is directly related to the adhesion force and energy.

### Adhesion force and energy measurement of a cell population using the calibrated biosensor

Once the quantitative relationship between the mechanical adhesion parameters and the biosensor signal (IWS for a given cell) is determined, we are able to measure the biologically relevant adhesion force on a large population of cells with the label-free imager biosensor. Following the previously described protocol, a population of HeLa cells was deposited on the RGD-functionalized biosensor surface and their adhesion kinetics was followed for a period of 90 minutes. At any time point the IWS of individual cells can be automatically calculated using the interpolation and Voronoi tessellation based approach defined previously. From the IWS values we can readily calculate the adhesion force using our calibration:

3 $$F=IWS\cdot (4.9\pm 0.5)\cdot {10}^{-4}\frac{nN}{pm\cdot \mu {m}^{2}}$$

The adhesion energy can be determined as

4 $$E=IWS\cdot (6.4\pm 0.7)\cdot {10}^{-6}\frac{pJ}{pm\cdot \mu {m}^{2}}$$

The throughput of the method is unprecedented. The optical biosensor collects signal from twelve sensor surfaces each with an area of 2 mm × 2 mm, which accounts for an overall sensitive area of 48 mm^2^. By assigning a 4000 µm^2^ average area to a single-cell, ideally, one can simultaneously measure the real-time adhesion force kinetics of 1200 cells. As a comparison, measuring the adhesion force of the same number of single-cells would take around 120 working days using an AFM, not to mention the astronomical costs of such an endeavor. Note, there is no theoretical limitation to increase the sensitive area of the RWG sensors, thus the described method is scalable.

**Figure 5:**
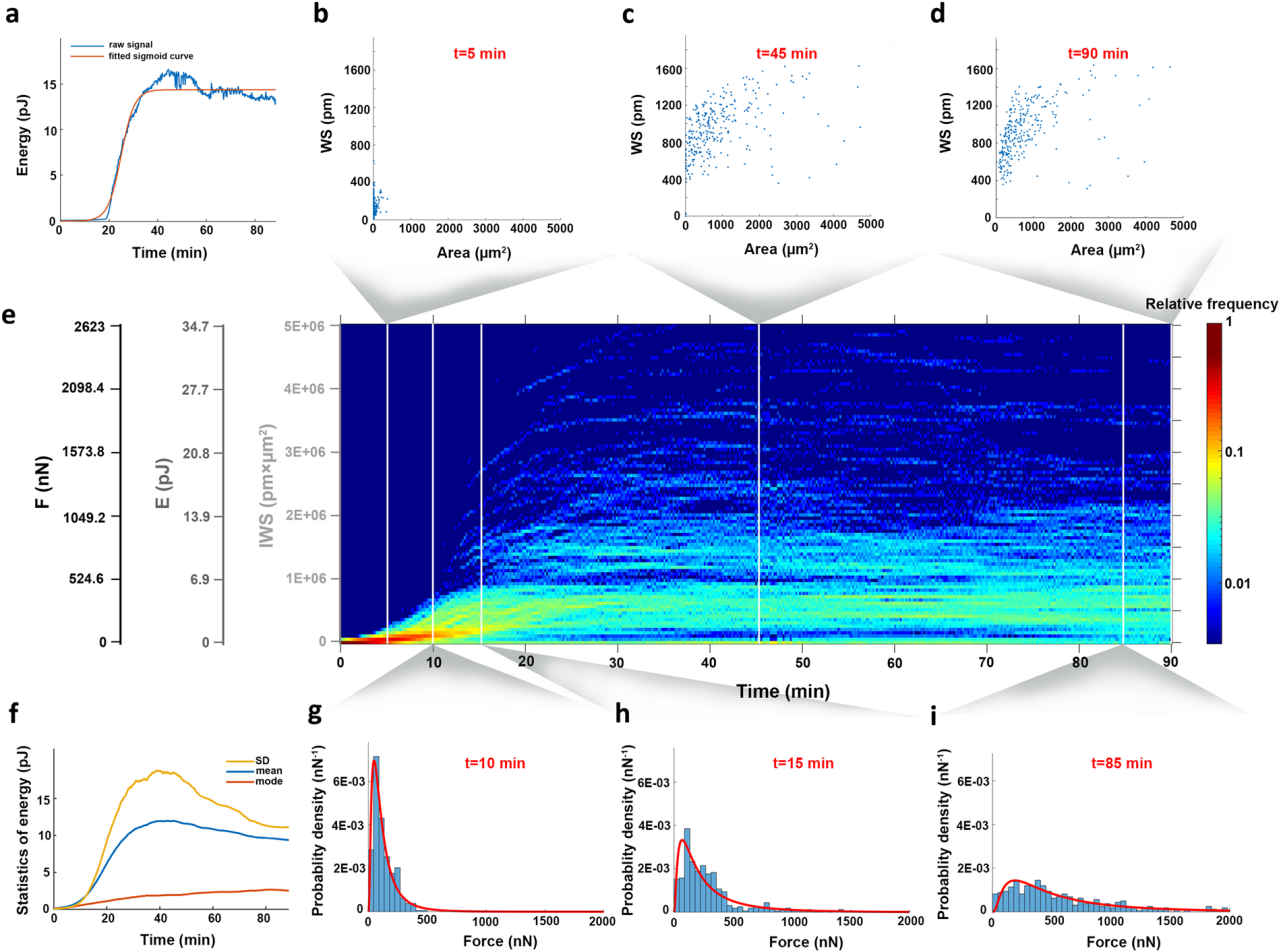
Single-cell level high-throughput adhesion force and energy results of large cell population obtained by the calibrated biosensor (**a**) A representative example of single-cell adhesion energy-time evolution (blue line) with its corresponding fitted sigmoidal curve (red line). (**b**–**d**) Time evolution of wavelength shift (WS) (averaged over the cell area) and cell area parameter state-space: after the initial phase of the spreading (**b**) the cells populate a larger area of the state space (**c**), then they reach the final , focused’ distribution (**d**). (**e**) Adhesion spectrogram: the representation gives insight to the real-time single-cell level statistics of a typical measurement. The color bar indicates the relative frequency on a logarithmic scale of a given bin value of force, energy or IWS (interpolation degree k = 0). (**f**) Time evolution of the adhesion energy statistics measures: at each timestep a log-normal distribution is fitted to the adhesion force distribution, and the derived mean (blue), mode (red) and variance (yellow) parameters are shown. Analogous to previous population level cell adhesion studies on wavelength shift, the mean of the adhesion energy shows a sigmoidal shape time dependence. (**g**,**h**,**i**) Representative time instances of the adhesion spectrogram: probability density histograms (blue) and their fitted log-normal probability density functions (red) of the adhesion force are shown at t = 10 min, 15 min and 85 min respectively.

The time-dependent signal of the label-free biosensor can be converted to a kinetic curve of adhesion energy for each single-cell on the basis of the FluidFM calibration (Fig. 5a). The curve can be fitted with a sigmoid function with typical parameters of 0.005 1/s as rate constant of spreading and 15 pJ as saturation value. The adhesion process of the entire population can be followed on a 2D scatter plot (Fig. 5b–d), where each dot represents a single cell in the cell averaged biosensor signal (WS) - contact area space. Figure 5e shows the real-time adhesion force/energy kinetics of more than 300 individual cells in an adhesion spectrogram visualization highlighting both population level kinetics and subpopulation level deviations. Cells with high adhesion force are relatively rare, but their contribution to the overall behavior of the population is significant. Thus measurements on a low number of cells can be misleading. Average adhesion energy of single-cells on the RGD-functionalized surface grows steadily in the first ~40 min of the adhesion process (Fig. 5f). Spreading of cells is accompanied by an increase of the standard deviation (SD) in the adhesion energy of individual cells. Analyzing the data, we found that the distribution of the adhesion force of single-cells can be well-fitted with a log-normal function, shown for three subsequent time points (see Fig. 5g–i). Parameters of the log-normal distribution as a function of time are shown in Fig. 5f. Note, log-normal distribution is general in natural phenomena^80–83^. In this specific case the long tail of the distribution means that extremely strongly attached cells are more frequent than it would be expected according to a Gaussian distribution. Furthermore, the initial widening and later narrowing distribution well demonstrates the dynamic changes in the heterogeneity of the population during the adhesion process.

## Discussion

The aim of the presented work was to demonstrate that the RWG-based biosensor can be used to measure single-cell level adhesion force and energy values en masse. Since the adhesion complexes formed in the close vicinity of the cell-surface contact area shift the local index of refraction, we assumed the WS signal (obtained by the biosensor) to be proportional to the mechanically measured adhesion characteristics.

In order to obtain an effective spatial characterization of the cells, we defined a surface-integrated version of the wavelength shift (IWS) provided by the Epic Cardio instrument. Suitably for the high throughput of the measurements, we developed an evaluating pipeline software, which can readily extract single-cell IWS signal from the raw, whole sensor WS image data. According to the presented results not only can we state that the IWS signal correlates with the adhesion force and energy values of the cells but we can also give an explicit conversion between the two numbers with a reasonable margin of error, being (4.9 ± 0.5) × 10^−4^ nN/(pm·µm^2^) for the force and (6.4 ± 0.7) × 10^−6^ pJ/(pm·µm^2^) for the energy values.

We established a rich database of single-cell adhesion kinetics on the basis of ~300 cells. HeLa cells show a high level of heterogeneity in the adhesion process described by three parameters, such as saturation value of the adhesion force, maximum speed of the adhesion process and final cellular area. We plan to build a single-cell adhesion database of many different cell types using the same biosensor and potentially different functionalized surfaces. Such experimental data are expected to enhance our understanding of the heterogeneity of single-cell phenotypes.

Our method can monitor different cell types on various surfaces in a single run. Our workflow beginning with biosensor surface functionalization and cell seeding can be fully automated to record and analyze single-cell adhesion data.

Consequently, the calibrated RWG-based biosensor can be considered the fastest existing cell adhesion force measurement method, unrivalled by conventional AFM or even by the FluidFM technique. Since the RWG technique very precisely measures the refractive index close to the substrate with a resolution of 10^−5^ refractive index unit^84^, it should be pointed out that the refractive index of the cells depends on the cell cycle, the current metabolic state and the type of the cell etc. The variation of these factors should be taken into account during the interpretation of the results. Nonetheless, estimating a hundred cells per sensor area, it is possible to simultaneously measure the adhesion force of 1200 cells on the 12 sensors that can be monitored by the device, in real-time. By further development of the plate-based instrument, the number of detected cells can be increased considerably without the need to extend the duration of the experiment. The inevitable cost of such a high throughput is a loss of accuracy. While the optical signal strongly correlates with the adhesion force, as shown in this study, unlike AFM based methods it is providing an indirect adhesion force value. (In cases where the complete adhesion force of a particular single cell has to be determined precisely, AFM and FluidFM remain the state of the art methods).

Nevertheless, using the RWG technique to measure cell adhesion forces comes with all the inherent advantages of optics-based methods: small size, convenient operation and scalability, to mention the most important ones. The ability to monitor the adhesion force spectrum of a large cell population in real-time opens up the possibility to study the effect of drugs on cell adhesion with single-cell accuracy and facilitate the development of a new class of cell-on-a-chip type biosensors. Measuring the response of a large number of individual cells in lieu of averaging over the population gives us the ability to study rare cell types and population inhomogeneity with respect to adhesion force and energy values.

Moreover, the present calibration methodology can be easily employed to other surface sensitive label-free biosensor devices as well. Considering these advantages, we expect that high spatial and temporal resolution surface sensitive label-free biosensors, especially the RWG-based technique, will become standard, widely used methods both in basic and applied research to measure cell adhesion force on a single-cell level with extremely high throughput.

## Methods

### Epic Cardio high-resolution label-free optical biosensor

The Epic Cardio imager biosensor (Corning Inc., Corning, NY, USA) used in our experiments accepts 384-well Society for Biomolecular Screening (SBS) standard format biosensor microplates. The plate is illuminated from below and the light is coupled into the thin high refractive index waveguide layer. Inside the layer, light propagation happens through a series of total internal reflections where each reflection adds a shift to the phase of the light beam. These phase changes create a constructive interference only for a specific wavelength component of the illuminating light, which in turn will be able to propagate in the waveguide for a short distance. The same grating used for incoupling will also couple the light with the resonant frequency out of the waveguide. This way the sensor area effectively becomes a monochromatic mirror, reflecting only a particular resonant component of the illumination. The sensing principle is based on the fact that the above mentioned phase shift that occurs during total internal reflection events is dependent on the refractive index (RI) of the close vicinity of the surface of the waveguide. This sensitive zone is determined by the extent of the evanescent field which is the exponentially decaying electromagnetic field generated when the light is reflected from the waveguide-aqueous cover boundary (Fig. 1a). As the RI changes in this area, the resonant frequency is altered through the change in the phase shift that the light acquires during total internal reflection. The outcoupled light and its wavelength is detected by a complementary metal-oxide semiconductor (CMOS) camera with a spatial resolution of 25 µm and a sampling time of 3 seconds.

### Robotic fluidic force microscopy and calibration of probes

Before cell adhesion measurements, the FluidFM BOT micropipette probe (Cytosurge AG) was placed into the robotic fluidic force microscopy device (FluidFM BOT, Cytosurge AG) for calibration. Probes with an aperture diameter of 8 µm and a nominal spring constant of 2 N/m were used. The fluidic channel of the probe was filled with 1 µl of 20 mM HBSS-HEPES (20 mM 4-(2-hydroxyethyl)-1-piperazineethanesulfonic acid (HEPES, Sigma-Aldrich Chemie GmbH, Schelldorf, Germany) in Hank’s Balanced Salt Solution (HBSS, Sigma-Aldrich Chemie GmbH)) buffer using a hand held pipette. Afterwards, the probe was mounted onto the head of the FluidFM and the laser was positioned at the end of the cantilever manually. The mirror reflecting the laser light to the position-sensitive detector (PSD) was automatically adjusted to achieve optimal light distribution between the sensor segments. The spring constant was measured using the thermal vibration spectrum of the cantilever (Sader method). Next, the probe was approached to the sample and the inverse optical lever sensitivity was measured by approaching the cantilever to the surface three times and fitting the deflection-displacement curve with a first order polynomial. The average of the three fitted slopes was accepted as the valid sensitivity (in a unit of m/V). The adhesion force curves were calculated by multiplying the differential signal of the PSD (measured in mV) with the sensitivity then with the spring constant to convert the distance values to force. The recorded force curves were then evaluated according to the standard protocol in literature^85,86^.

### Measurement chamber for combined experiments and related protocols

Since the standard 384 well of the biosensor microplate is far too small for the FluidFM BOT probe to reach the adhered cells, we used 384-well uncoated Epic microplates (Corning Inc.) without the plastic wells glued on the glass substrate containing the waveguide sensors (insert). In order to allow for easy navigation with the FluidFM probe in the second stage of the experiment, we created a custom plate holder that contains two circular wells each with a 3 cm diameter (Figs. 1b,c and 3d). A biosensor insert was placed between the lower and upper parts of the holder and fixed by screwing the parts together. The biosensor device (Fig. 1c) used in this study can detect 12 of the 2 mm by 2 mm sensor surfaces contained within one of the circular wells. After the coating of the well with cell adhesion inducing polymers (see next paragraph for details), the holder was placed onto the device and its position was adjusted using positioning screws. Once the sensor surfaces were aligned to overlap the area detected by the CMOS camera inside the biosensor device, a baseline measurement was started.

To prepare the combined experiment, cells were washed with Dulbecco’s phosphate-buffered saline (DPBS, Sigma-Aldrich Chemie GmbH), and detached with 10 mM EDTA for 10 min in an incubator (37 °C, 5% CO_2_). The cell pellet was resuspended in assay buffer and diluted to ideal concentration for the experiments. The cells were added to the well containing assay buffer after 30–40 min of baseline recording and they were let to adhere on the sensor for 90 min. Afterwards, the measurement was stopped and the plate was placed into the sample holder of the already calibrated FluidFM BOT device. Cell adhesion measurements were continued for about two hours while the cells were still alive. In order to identify the same cells in the optical microscope of the FluidFM BOT that are visible on the image generated by the Epic Cardio biosensor, we related the position of the cell to the scratch marks on the sensor surface that were introduced before the coating procedure. Once a cell was identified we proceeded to detach it with the FluidFM probe.

### Detachment protocols of cells in FluidFM BOT

After the calibration of the FluidFM probe and subsequent placement of the custom plate into the sample holder, individual cells were targeted for detachment. Cells were approached from a height of ~20 µm using a setpoint of 20 mV, which defines the threshold value of the FluidFM signal considered to represent the mechanical contact with the cell surface. When the setpoint was reached, −300 to −700 mbar negative pressure was applied in the cantilever, the exact value being determined according to cell size and morphology. The cantilever was kept in contact with the cell for 15 s then it was retracted to a height of 70 µm with a retraction speed of 1 µm/s. The force-distance curves were then analyzed for adhesion strength and energy. The force value at the last point of the retraction was accepted as 0 nN and the curve was shifted along the y axis accordingly. The absolute minimum was considered to be the adhesion force, while the integral between the zero points was taken as the adhesion energy.

### Preparation of biosensor coatings

In the middle of one of the custom wells, a rubber insert was placed to minimize reagent usage. Around 1 mm long scratch marks were introduced into the surface with a diamond head glass cutter to facilitate the later identification of the cells. To induce cell adhesion in a controllable manner, we used a mixture of poly(L-lysine)-*graft*-poly(ethylene glycol) (PLL-*g*-PEG, [PLL(20)-*g*(3.5)-PEG(2)]) (hereafter PP) and its RGD-functionalized counterpart: PLL-*g*-PEG/PEGGGGGYGRGDSP (PLL-*g*-PEG-RGD-12% [PLL(20)-*g*(3.5)-PEG(2)/PEG(3.4)-RGD]) (hereafter PPR). Both materials were obtained as powder from SuSoS AG (Dübendorf, Switzerland) and were stored at −20 °C until use. The well was filled with a coating solution consisting of 1–50% PPR to PP dissolved in 10 mM pH 7.4 HEPES buffer (coating buffer) at 0.5 mg/ml concentration. The plate was then placed on a rocker for 30 min at room temperature to allow for the adsorption of the polymers to the surface. Afterwards, the coating solution was removed and the well was washed two times with the coating buffer which was then replaced by 2 ml of the assay buffer HBSS containing 20 mM HEPES (HBSS- HEPES), pH 7.4).

### Cell culture

HeLa cell line (93021013 Sigma-Aldrich) was maintained in Dulbecco’s Modified Eagle’s Medium (DMEM), supplemented with 10% fetal bovine serum (Biowest SAS, France), 4 mM L-glutamine, 100 U/mL penicillin and 100 µg/mL streptomycin solution. Cells were cultured in a humidified atmosphere containing 5% CO_2_ at 37 °C.

## Supplementary information

Supplementary Figure

## Data Availability

All analyzed data of this study are contained within the manuscript.
