# Supplementary material for: Single-cell adhesion force kinetics of cell populations from combined label-free optical biosensor and robotic fluidic force microscopy
Source: Sci Rep. 2020 Jan 9;10:61. doi: 10.1038/s41598-019-56898-7 (PMC6952389; doi:10.1038/s41598-019-56898-7)
Supplement: Supplementary file 1 — Supplementary Figure [file 41598_2019_56898_MOESM1_ESM.docx]

**Supplementary Information**

**Single-cell adhesion force kinetics of cell populations from combined label-free optical biosensor and robotic fluidic force microscopy**

***Milan Sztilkovics ^a,*^, Tamas Gerecsei ^a,b,*^, Beatrix Peter ^a^, Andras Saftics ^a^, Sandor Kurunczi ^a^ , Inna Szekacs ^a^, Balint Szabo ^b^ and Robert Horvath ^a,**^***

^a^ Nanobiosensorics Group, Institute of Technical Physics and Materials Science, Centre for Energy Research, Budapest, Hungary

^b^ Department of Biological Physics, Eötvös University, Budapest, Hungary

*Equal contribution

**Correspondence to r74horvath@gmail.com

During the detachment process, an adhesion force is acting between the solid substrate and the cell membrane. The FluidFM cantilever overcomes this force as it separates the cell from the surface. Ideally, the part of the cell membrane that is initially in contact with the surface will stay physically intact during this process. This means that there should be no holes, fissures or perforations on the membrane that would compromise their topological integrity. This way, the force measured by the cantilever exclusively originates at the membrane-surface contact and does not include any force components coming from a certain tearing or rupturing of the membrane. Therefore, in these cases we expect to see no remnants of the membrane left behind on the substrate after detachment (Figure S1 a-b).

On the other hand, in certain cases it might happen that the membrane is ripped during detachment, leaving behind fragments that are visible as dark blobs remaining after the cell is removed from the surface (Figure S1 c-d). In such cases, the adhesion measurement was not accepted for evaluation, since the tearing up of the membrane influences the adhesion curve.


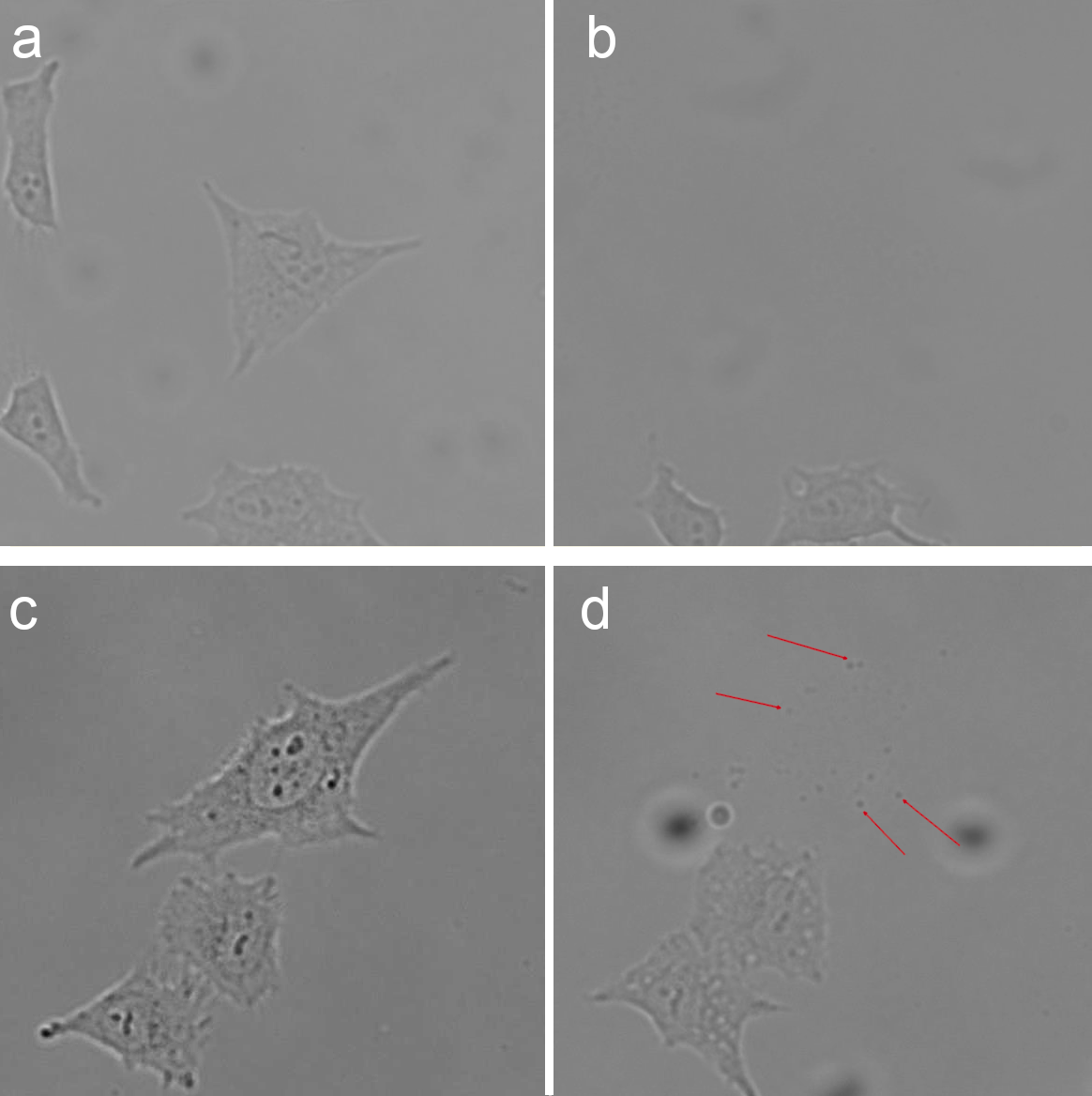


S 1: Figure showing the surface after detachment of cells using FluidFM. All images were made in bright field mode using an inverted microscope (Zeiss Axio Observer Z1). **a-b** Image of the surface before and after cell detachment with the FluidFM. There are no visible signs of any debris that would indicate a damaged membrane. Such detachment events were accepted for evaluation. **c-d** Image of the surface before and after cell detachment with the FluidFM. There is visible debris left by the cell (pointed out by red arrows) which hints at physical membrane damage. Such measurements were not accepted for evaluation.
